# Association between heavy metals exposure (cadmium, lead, arsenic, mercury) and child autistic disorder: a systematic review and meta-analysis

**DOI:** 10.3389/fped.2023.1169733

**Published:** 2023-07-04

**Authors:** Mengmeng Ding, Shanshan Shi, Shuyan Qie, Jinglu Li, Xiaoming Xi

**Affiliations:** Rehabilitation Treatment Center, Beijing Rehabilitation Hospital Affiliated to Capital Medical University, Beijing, China

**Keywords:** autism spectrum disorder, trace elements, cadmium, lead, arsenic, mercury

## Abstract

**Background:**

Studies have found that toxic heavy metals exposure could induce the generation of reactive oxygen species (ROS), and is of epigenetic effect, which might be associated with the occurrence of Autistic Disorder (ASD). This systematic review and meta-analysis aims to elucidate the association between exposure to 4 heavy metals, cadmium (Cd), lead (Pb), arsenic(As), and mercury (Hg), and the occurrence of ASD in children.

**Methods:**

We searched PubMed, Web of Science, Embase, and Cochrane Library, from their inception to October 2022, for epidemiological investigations that explore the association between exposure to Cd, Pb, As, or Hg and the occurrence of child ASD.

**Results:**

A total of 53 studies were included, involving 5,054 individuals aged less than 18 (2,533 ASD patients and 2,521 healthy controls). Compared with the healthy controls, in hair and blood tests, concentrations of the 4 heavy metals were significantly higher in the ASD group than in the healthy control group, and the differences in Pb, arsenic and Hg were statistically significant (*P* < 0.05). In the urine test, concentrations of arsenic and Hg were significantly higher in the ASD group than in the healthy control group (*P* < 0.05), while the results of Cd and Pb were opposite to those of arsenic and Hg (*P* > 0.05). Subgroup analysis for geographic regions showed that ASD patients in Asia and Europe had higher concentrations of the 4 heavy metals, compared with the healthy controls, in which the differences in Pb, arsenic, and Hg were statistically significant (*P* < 0.05), while in North America, the healthy controls had higher Cd, arsenic, and Hg concentrations (*P* > 0.05).

**Conclusion:**

Compared with the healthy control group, the ASD group had higher concentrations of Cd, Pb, arsenic, and Hg. These 4 heavy metals play different roles in the occurrence and progression of ASD. Moreover, there is significant heterogeneity among the included studies due to controversies about the study results among different countries and regions and different sources of detection materials. The results of this study firmly support the policies to limit heavy metals exposure, especially among pregnant women and young children, so as to help reduce the incidence of ASD.

## 1. Introduction

Autistic Disorder (ASD) refers to a complex neurodevelopmental disorder affecting approximately 78 million people worldwide. The morbidity of ASD has been rapidly increasing during the past decades, and its overall estimated prevalence ranges from 1.5% to 2% (1, 2). Despite the rapid increase in its morbidity, there remains a poor understanding of the pathogenesis, risk factors, and prognosis of ASD. There are neither biomarkers that can be applied for disease screening and diagnosing, nor effective therapeutic agents (1). ASD presents to be a lifelong disease bringing a heavy burden on both the patients’ family and social health care system, making the prophylaxis of ASD an important public health problem.

Recent studies have shown that the pathogenesis of ASD would be multi-factorial, with genetic, biophysiological, and environmental factors (such as heavy metals exposure) jointly involved. Environmental factors (including neurotoxic heavy metals exposure) play a critical role in the occurrence and progression of ASD. Several studies discover that among the risk factors for ASD, environmental factors are more important than genetic factors (3). Mohamed et al. have found that exposure to heavy metals during pregnancy, consumption of chemically-contaminated foods, resident near petrol stations, and use of aluminum-made pots are associated with the occurrence of ASD (4). Dickerson et al, have found that urban populations residing near industrial facilities that discharge air pollutants and heavy metals (arsenic, Pb, and Hg) have a higher incidence of ASD (5). In addition, multiple studies also indicate an association between heavy metal-related biomarkers and ASD occurrence (3, 6–8).

Heavy metals and their compounds exist widely in the natural environment. These substances are difficult to be metabolized due to their stable chemical properties, and can accumulate in the food chain. Previous epidemiological investigations show that heavy metal exposure plays a significant role in the pathogenesis of ASD. Exposure to these toxic substances induces the release of cytotoxic substances, immune response, neuronal inflammation, and generation of reactive oxygen species (ROS), and subsequently causes irreversible damage to the brain development of ASD individuals (9–11). However, there are some other studies that have observed no significant differences in the severity of heavy metals exposure between ASD patients and healthy populations. The association between heavy metals exposure and ASD occurrence remains controversial. This study, through a comprehensive literature search, selects 4 heavy metals (Cd, Pb, arsenic, Hg) that are well-studied and cause wide controversies in the field of ASD to perform a systematic review and meta-analysis.

There is just 1 meta-analysis regarding the association between heavy metal exposure and ASD (12), which included 22 studies, 18 microelements, and 2,013 participants, while the study design was simple and the literature search was uncomprehensive. We selected 4 controversial heavy metals, included 53 studies and 5,054 participants, and performed meta-analysis based on case-control studies, so as to identify the association between heavy metals exposure and ASD occurrence and provide a reference for the prevention of child ASD.

## 2. Methods

### 2.1. Search strategy

PubMed, Web of Science, Embase, and Cochrane Library were searched, from their inception to October 2022, for studies regarding the association of Cd, Pb, arsenic, and Hg exposure with the occurrence of ASD. Search items mainly included: “Autistic Disorder” OR “Autism” AND “Cadmium” OR “Lead” OR “arsenic” OR “mercury”. The detailed search strategy is provided in Supplementary Files S1.

### 2.2. Inclusion and exclusion criteria

#### 2.2.1. Inclusion criteria

• ASD patients aged less than 18.
• Case-control study taking ASD children as observation group and healthy populations as control.
• Tested types of heavy metals should include at least one of the above-mentioned heavy metals.

#### 2.2.2. Exclusion criteria

• Letter, conference summary, case report, literature review, animal experiment, in-vitro or laboratory study, and studies published in non-English languages.
• Study with the data of full-text unavailable.

### 2.3. Date extraction

After searching all the databases, data extraction was performed on the included articles, including publication time, number of included participants, geographical location of included population, types of heavy metals for detection, sources of detection substances, detection methods, mean and standard deviation of heavy metal concentrations.

### 2.4. Quality assessment

We adopted the New Castle-Ottawa Scale (NOS) to assess the methodological quality of included studies, which contains 3 domains: Selection, Comparability, and Outcome. Each study can be scored for at most 9, studies scored over 6 would be graded as of high quality (13). Two researchers independently conducted the assessment, and disagreements were settled by discussion.

### 2.5. Statistical analysis

Statistical analyses were performed using Stata 15.0 (StataCorp, College Station, TX, USA). Heterogeneity among the included studies was assessed by the Cochrane Q test and *I*^2^ statistic. If an *I*^2^ was less than 50%, a fixed-effects model was applied to estimate the effect size and the 95% confidence interval (95%CI); otherwise, a random-effects model would be used. Continuous data were expressed as Standardized Mean Difference (SMD). Since the included literature was from different countries and regions, and different researchers have not uniformly selected the detection substances for detection of heavy metals, we conducted subgroup analyses on the participants' countries and sources for heavy metal detection, to further explore the reasons for the difference in heavy metal concentration between the ASD group and the control group. Sensitivity analysis was performed to assess the robustness of the results, by removing the included studies one by one to detect potential outliers. Publication bias was assessed using Egger’s and Begg’s tests. A *P*-value greater than 0.05 indicated no evident publication bias; otherwise, the Trim-and-Fill method would be used to detect the small-sample effect. A *P*-value less than 0.05 would be considered statistically significant.

## 3. Results

### 3.1. Study selection and basic characteristics

**Figure 1:**
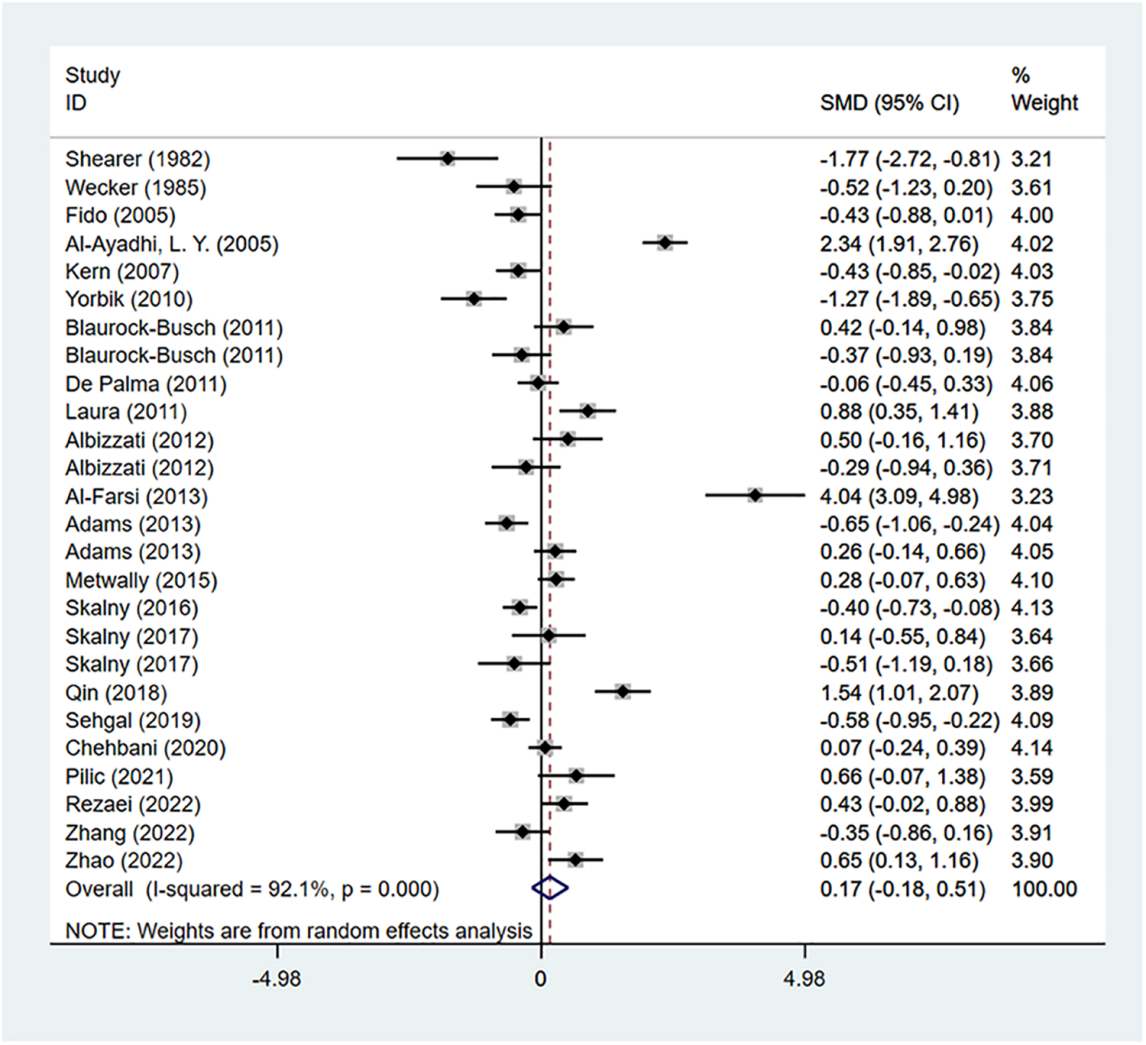
Flow chart of literature screening.

A total of 1,374 articles were retrieved (372 from PubMed, 341 from Embase, 645 from Web of Science, and 16 from Cochrane), and 574 duplicates were removed. Guideline (15 studies), case report (28 studies), letter (20 studies), review (110 studies), animal experiments (44 studies), meta-analysis (29 studies), irrelevant to trace elements and ASD (465 studies), full text not available (7 studies), and unretrievable data (29 studies) were excluded through browsing titles and abstracts. Finally, 53 studies (14–64) were included (Table 1 and Figure 1), involving 5,054 participants (2,533 ASD children and 2,521 healthy controls). Among the included studies, 12 studies were conducted in North America, 19 were conducted in Asia, 20 in Europe, 1 in Australia, and 1 in Africa.

**Table 1:** Basic information of the literature was included.

First author	year	Country	Sample size	age	Metals	Detection source	quality
Shearer	1982	America	ASD:12 Control:12	2–16	Cd; Pb	hair	7
Wecker	1985	America	ASD:12 Control:22	4–6	Cd; Pb; Hg	hair	7
Holmes	2003	America	ASD:94 Control:45	2–15	Hg	hair	6
Ip	2004	Hong Kong	ASD:82 Control:55	7–8	Hg	Hair; blood	8
Fido	2005	Kuwait	ASD:40 Control:40	4–8	Cd; Hg; As; Pb	hair	7
Al-Ayadhi	2005	Saudi Arabia	ASD:65 Control:80	6–16	Cd; Hg; As; Pb	hair	6
Adams	2007	England	ASD:15 Control:11	4–8	Hg; Pb	teeth	7
Kern	2007	America	ASD:45 Control:45	1–6	Cd; Hg; As; Pb	hair	7
Williams	2008	America	ASD:15 Control:16	2–6	Hg	hair	7
El-baz	2010	Egypt	ASD:32 Control:15	2–13	Hg	hair	7
Geier	2010	America	ASD:83 Control:89	3–14	Hg	blood	7
Woods	2010	America	ASD:64 Control:114	3–9	Hg	urine	6
Yorbik	2010	Turkey	ASD:30 Control:20	3–12	Cd; Pb	urine	7
Blaurock-Busch	2011	Germany	ASD:25 Control:25	3–9	Cd; Hg; As; Pb	Urine; hair	6
De Palma	2011	Italy	ASD:44 Control:61	2–17	Cd; Hg; As; Pb	Hair; blood; urine	7
Lakshmi Priya	2011	India	ASD:45 Control:50	4–12	Hg; Pb	Hair; nails	7
Laura	2011	Italy	ASD:28 Control:32	2–6	Cd; As; Pb	blood	7
Abdullah	2012	America	ASD:22 Control:22	9–14	Pb; Hg	teeth	7
Albizzati	2012	Italy	ASD:17 Control:20	6–16	Cd; Hg; Pb	urine	7
Wright	2012	Australia	ASD:54 Control:121	6–14	Hg	urine	7
Adams	2013	America	ASD:54 Control:44	5–16	Cd; As; Pb	Blood; urine	6
Al-Farsi	2013	Oman	ASD:27 Control:27	2–17	Cd; Pb	hair	7
Alabdali	2014	Saudi Arabia	ASD:58 Control:32	3–12	Hg; Pb	blood	7
Yassa	201	Egypt	ASD:45 Control:45	2–10	Hg; Pb	Blood; hair	7
Yau	2014	America	ASD:70 Control:128	3–4	Hg	blood	7
Fuentes-Albero	2015	Spain	ASD:35 Control:34	4–13	Pb	urine	6
Metwally	2015	Egypt	ASD:55 Control:75		Cd; Hg; As; Pb	hair	7
Mohamed	2015	Egypt	ASD:100 Control:100	2.5–15	Hg; Pb	hair	7
Rahbar	2015	AMerica	ASD:100 Control:100	2–8	Pb	blood	7
Khaled	2016	Egypt	ASD:40 Control:40	3–6	Hg; Pb	blood	7
Mostafa	2016	Egypt	ASD:84 Control:84	3–10	Hg	blood	7
El-Ansary	2017	Saudi Arabia	ASD:35 Control:35	3–12	Hg; Pb	blood	7
Gaza	2017	Indonesia	ASD:20 Control:20	5–17	Cd; Hg; Pb	hair	7
Skalny	2016	Russia	ASD:74 Control:74	2–9	Cd; Hg; As; Pb	hair	6
Skalny	2017	Russia	ASD:48 Control:48	3–9	As	blood	7
Skalny	2017	Russia	ASD:33 Control:33	3–8	Cd; Hg; As; Pb	hair	7
Qin	2018	China	ASD:34 Control:38	3–5	Cd; Hg; Pb	blood	6
Alawad	2018	Iraq	ASD:60 Control:47	0.3–13	Pb	blood	6
Sehgal	2019	India	ASD:60 Control:60	3–12	Cd; Hg; As; Pb	blood	7
Waligóra	2019	Poland	ASD:18 Control:20	2–15	Hg	Hair; urine	6
Chehbani	2020	Spain	ASD:89 Control:70		Cd; Hg; Pb	blood	7
Ali, A. T	2020	Egypt	ASD:20 Control:20		Pb	blood	6
Filon	2020	Poland	ASD:30 Control:30	2–8	As; Pb	hair	6
Gil-Hernández	2020	Spain	ASD:54 Control:5	2–6	Hg	Hair; urine	7
Hawari	2020	Syria	ASD:31 Control:30	3–12	Pb	blood	7
Pilic	2021	Bosnia and Herzegovina	ASD:16 Control:15	1–14	Cd; Pb	hair	6
Rashaid	2021	America	ASD:57 Control:50	4–12	Pb	hair	7
Abd Wahil	2022	Malaysia	ASD:81 Control:74	3–6	Pb	urine	7
Hegde	2022	India	ASD:70 Control:70		As	blood	7
Ouisselsat	2022	Morocco	ASD:107 Control:120	3–14	Pb; Hg	hair	7
Rezaei	2022	Germany	ASD:44 Control:35		Cd; As; Pb	urine	7
Zhang	2022	China	ASD:30 Control:30	2–6	Cd; Hg; Pb	blood	7
Zhao	2022	China	ASD:30 Control:30	2.5–5.7	Cd; Hg; As; Pb	blood	7

### 3.2. Quality assessment

The NOS scores of all the included studies were greater than 6, with an average score of 7.1(Table 1), which indicated no studies of low quality were included.

### 3.3. Association of CD exposure with ASD

**Figure 2:**
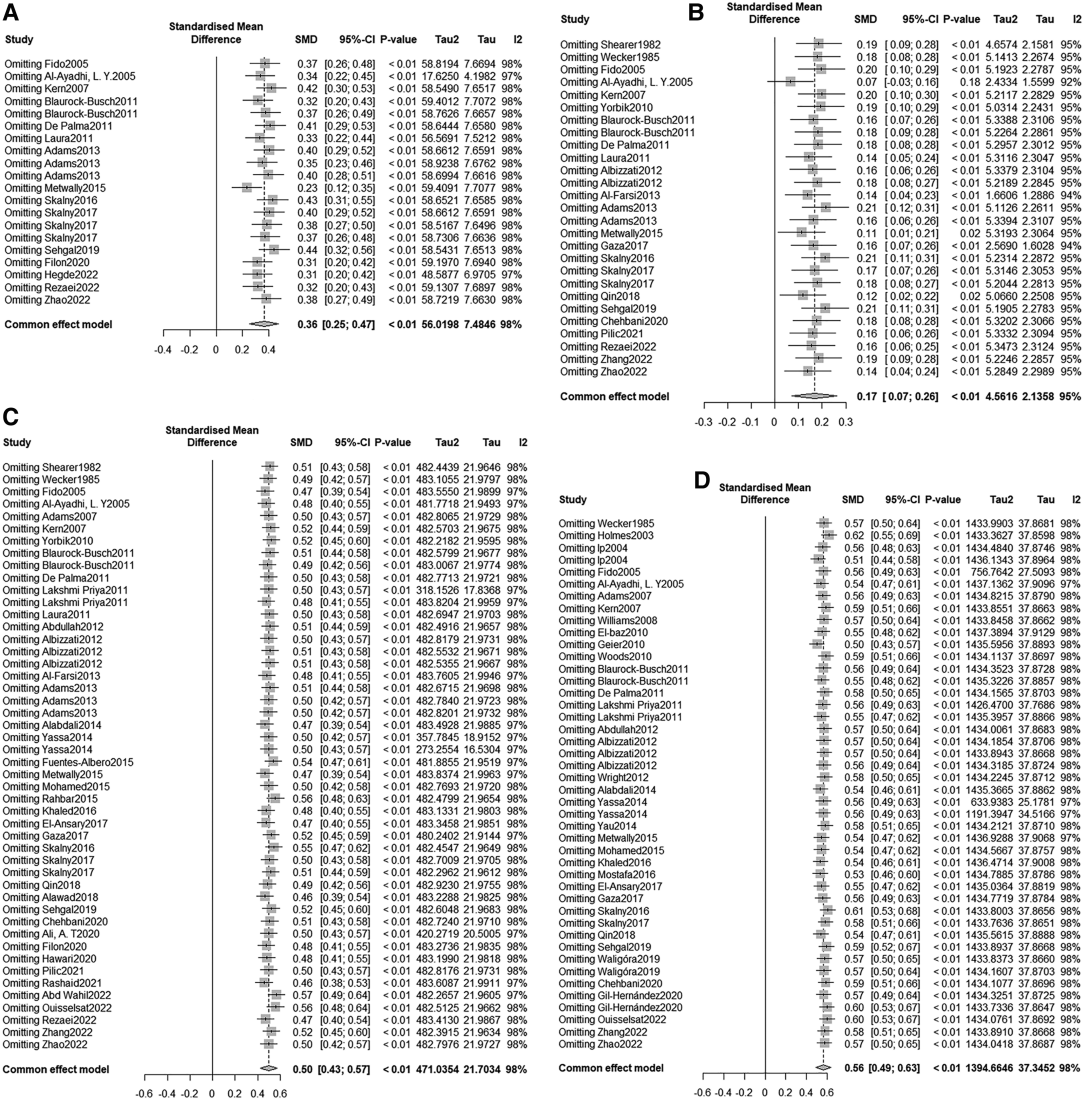
Cd meta-analysis forest plot.

**Figure 3:**
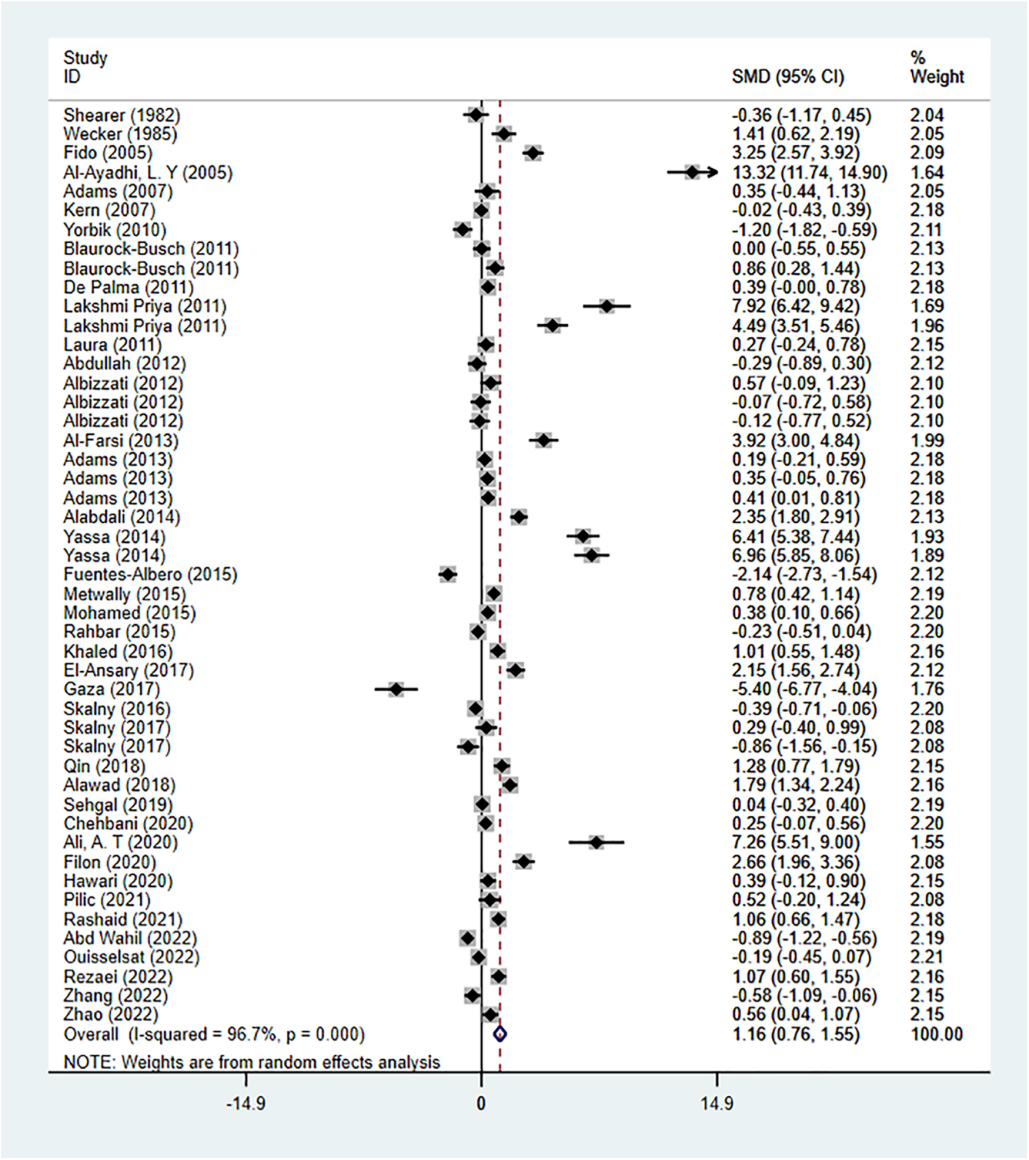
Sensitivity analysis (**A**) As (**B**) Cd (**C)** Hg (**D**) Pb.

There were 22 studies reporting the difference in Cd concentration between the ASD group and the healthy control group. A random-effects model was applied for meta-analysis (*I*^2 ^= 92.1%, *P* < 0.001). ASD group had higher Cd concentration compared to the healthy control group [SMD = 0.17, 95%CI (−0.18, 0.51), *P* > 0.05] (Figure 2). Subgroup analysis based on geographical regions of the participants and the testing sources showed that compared with the healthy controls, ASD patients had higher Cd concentration in hair [SMD = 0.22, 95%CI (−0.33, 0.77), *P* > 0.05] and blood [SMD = 0.21, 95%CI (−0.36, 0.77), *P* > 0.05], while had lower Cd concentration in urine [SMD = −0.05, 95%CI (−0.69, 0.59), *P* > 0.05]. As for geographical regions, ASD patients in Asia [SMD = 0.61, 95%CI (−0.18, 1.40), *P* > 0.05] and in Europe [SMD = 0.12, 95%CI (−0.18, 0.42), *P* > 0.05] had higher Cd concentration than the healthy controls in these regions, while patients in Australia [SMD = −0.52, 95% (−1.04, −0.01), *P* < 0.05] had a lower concentration (Table 2). Sensitivity analysis showed that the removal of the included studies one by one did not reverse the results, suggesting the results of the meta-analysis were robust. No significant publication bias was observed (*P* = 0.507 in Begg’s test and *P* = 0.67 in Egger’s test) (Figure 3).

**Table 2:** Subgroup analysis.

Subgroup analysis	Study number (n)	Model (Random effects model, REM)	SMD (95%CI)	*P* (*P*-value)	Heterogeneity	
					*I*^2^ (%)	*P_h_* (The *P*-value of heterogeneity)
**Cd**						
Area						
North America	5	REM	−0.52(−1.04, −0.01)	0.048	79.9	0.001
Asia	9	REM	0.61(−0.18, 1.40)	0.128	96.2	0.000
Europe	9	REM	0.12(−0.18, 0.42)	0.430	74.7	0.000
Detection source						
Hair	13	REM	0.22(−0.33, 0.77)	0.429	94.1	0.000
Urine	5	REM	−0.05(−0.69, 0.59)	0.879	83.9	0.000
Blood	7	REM	0.21(−0.36, 0.77)	0.476	94.1	0.000
**Pb**						
Area						
North America	7	REM	0.27(−0.08, 0.61)	0.130	81.4	0.000
Asia	16	REM	1.77 (0.85, 2.69)	0.000	98.0	0.000
Europe	17	REM	1.08 (0.53, 1.63)	0.000	96.1	0.000
Detection source						
Hair	20	REM	1.56 (0.85, 2.28)	0.000	97.5	0.000
Urine	7	REM	−0.28(−1.09, 0.53)	0.497	94.8	0.000
Blood	16	REM	1.23 (0.70, 1.77)	0.000	95.5	0.000
**As**						
Area						
North America	2	REM	−0.09(−0.33, 0.15)	0.464	30.2	0.231
Asia	7	REM	2.49 (1.07, 3.90)	0.001	98.5	0.000
Europe	6	REM	1.19 (0.40, 1.99)	0.003	94.1	0.000
Detection source						
Hair	9	REM	1.41 (0.45, 2.37)	0.004	97.4	0.000
Urine	3	REM	0.34 (0.00, 0.68)	0.051	38.8	0.198
Blood	6	REM	1.98 (0.59, 3.37)	0.005	98.3	0.000
**Hg**						
Area						
North America	8	REM	−0.02(−0.62, 0.59)	0.951	94.2	0.000
Asia	13	REM	1.55 (0.82, 2.29)	0.000	97.0	0.000
Europe	12	REM	0.55 (0.24, 0.86)	0.000	87.1	0.000
Detection source						
Hair	20	REM	0.91 (0.35, 1.48)	0.001	96.7	0.000
Urine	20	REM	0.29 (0.11, 0.47)	0.002	10.0	0.352
Blood	13	REM	0.77 (0.35, 1.19)	0.000	92.6	0.000

### 3.4. Association of Pb exposure with ASD

**Figure 4:**
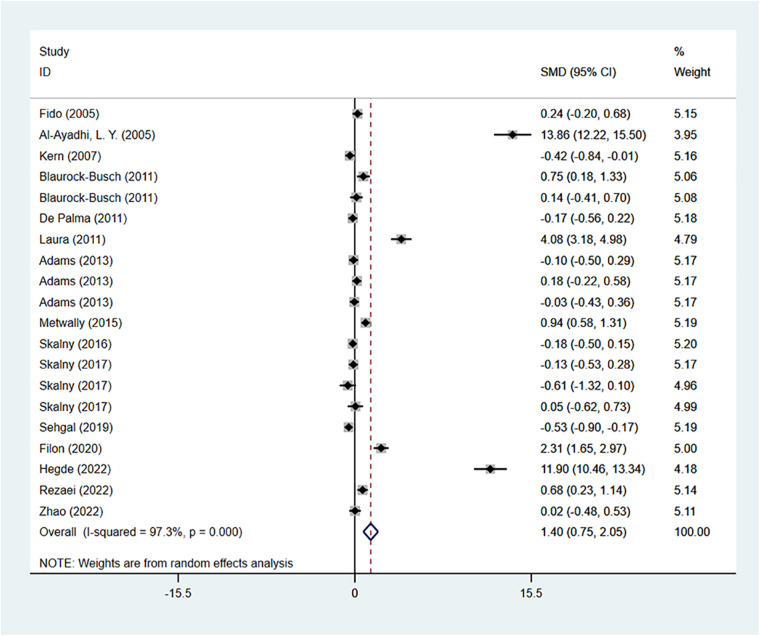
Pb meta-analysis forest plot.

There were 41 studies reporting the difference in Pb concentration between the ASD group and the healthy control group. A random-effects model was applied for meta-analysis (*I*^2 ^= 96.7%, *P* < 0.001). ASD group had higher Pb concentration compared to the healthy control group [SMD = 1.16, 95%CI (0.76, 1.55), *P* < 0.001] (Figure 4). Subgroup analysis based on geographical regions of the participants and the testing sources showed that compared with the healthy controls, ASD patients had higher Pb concentration in hair [SMD = 1.56, 95%CI (0.85, 2.28), *P* < 0.001], fingernails [SMD = 4.49, 95%CI (3.51, 5.46), *P* < 0.05], and blood [SMD = 1.23, 95%CI (0.70, 1.77), *P* < 0.001], while had lower Pb concentration in urine [SMD = −0.28, 95%CI (−1.09, 0.53), *P* > 0.05] and teeth [SMD = −0.03, 95%CI (−0.64, 0.59), *P* > 0.05]. As for geographical regions, ASD patients in North America [SMD = 0.27, 95%CI (−0.08, 0.61), *P* > 0.05], Asia [SMD = 1.77, 95%CI (0.85, 2.69), *P* < 0.001], and Europe [SMD = 1.08, 95%CI (0.53, 1.63), *P* < 0.001] had higher Pb concentration than the healthy controls in these regions (Table 2). Sensitivity analysis showed that the removal of the included studies one by one did not reverse the results, suggesting the results of the meta-analysis were robust. Significant publication bias was observed (*P* = 0.002 in Begg’s test and *P* = 0.000 in Egger’s test) (Figure 3). Trim-and-Fill method showed no newly-added studies, and no small-sample effect. Publication bias did not affect the results (Supplementary Files S2).

### 3.5. Association of arsenic exposure with ASD

**Figure 5:**
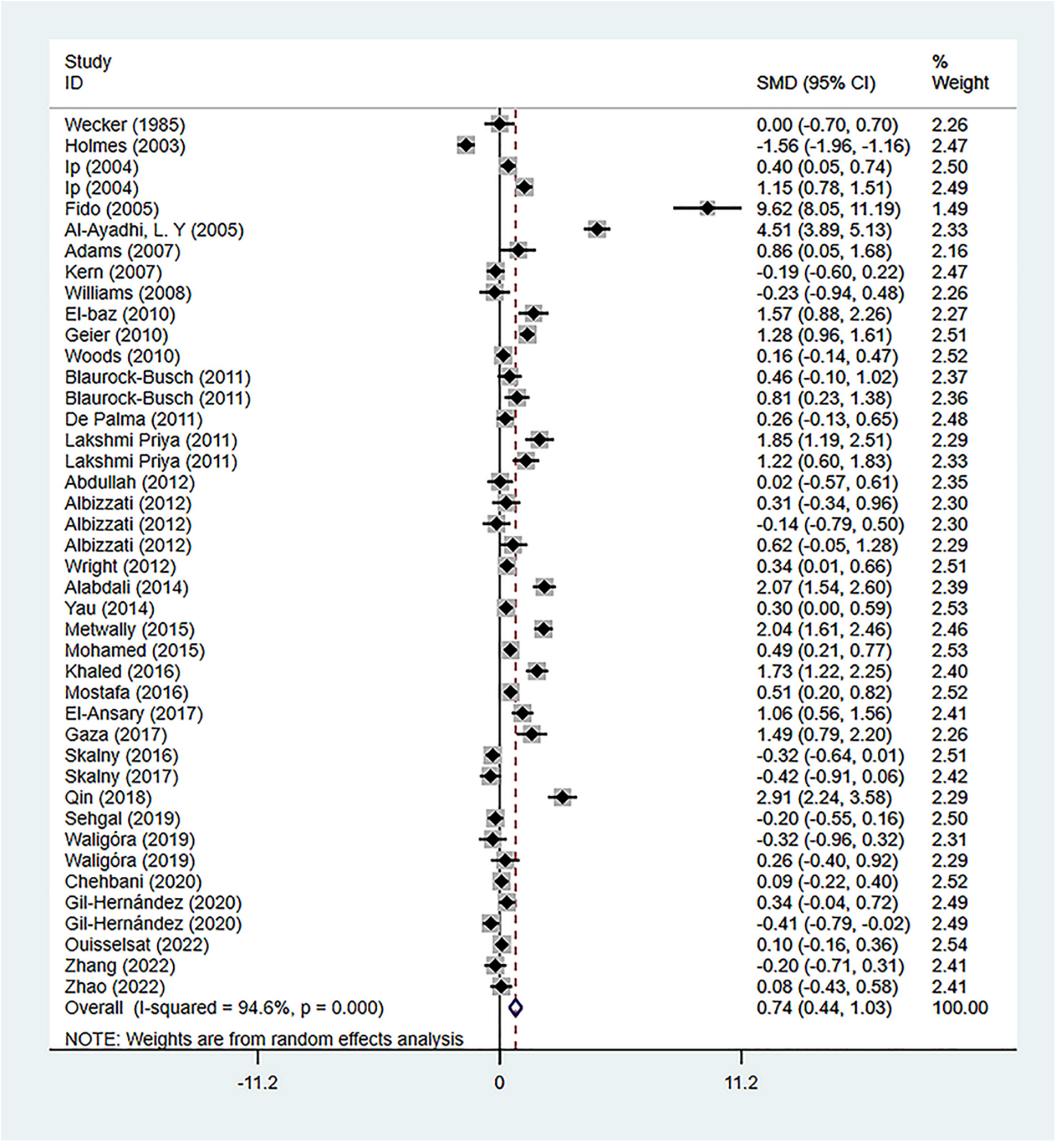
As meta-analysis forest plot.

There were 16 studies reporting the difference in arsenic concentration between the ASD group and the healthy control group. A random-effects model was applied for meta-analysis (*I*^2 ^= 97.3%, *P* < 0.001). ASD group had higher arsenic concentration compared to the healthy control group [SMD = 1.40, 95%CI (0.75, 2.05), *P* < 0.001] (Figure 5). Subgroup analysis based on geographical regions of the participants and the testing sources showed that compared with the healthy controls, ASD patients had higher arsenic concentration in hair [SMD = 1.41, 95%CI (0.45, 2.37), *P* < 0.05], urine [SMD = 0.34, 95%CI (0.00, 0.68), *P* > 0.05], and blood [SMD = 1.98, 95%CI (0.59, 3.37), *P* < 0.05]. As for geographical regions, ASD patients in Asia [SMD = 2.49, 95%CI (1.07, 3.90), *P* < 0.05] and Europe [SMD = 1.19, 95%CI (0.40, 1.99), *P* < 0.05] had higher arsenic concentration than the healthy controls in these regions, while patients in North America [SMD = −0.09, 95%CI (0.40, 1.99), *P* < 0.05] had a lower arsenic oncentration (Table 2). Sensitivity analysis showed that the removal of the included studies one by one did not reverse the results, suggesting the results of the meta-analysis were robust. Significant publication bias was observed (*P* = 0.003 in Begg’s test and *P* = 0.000 in Egger’s test) (Figure 3). Trim-and-Fill method showed no newly-added studies, and no small-sample effect. Publication bias did not affect the results (Supplementary Files S3).

### 3.6. Association of Hg exposure with ASD

**Figure 6:**
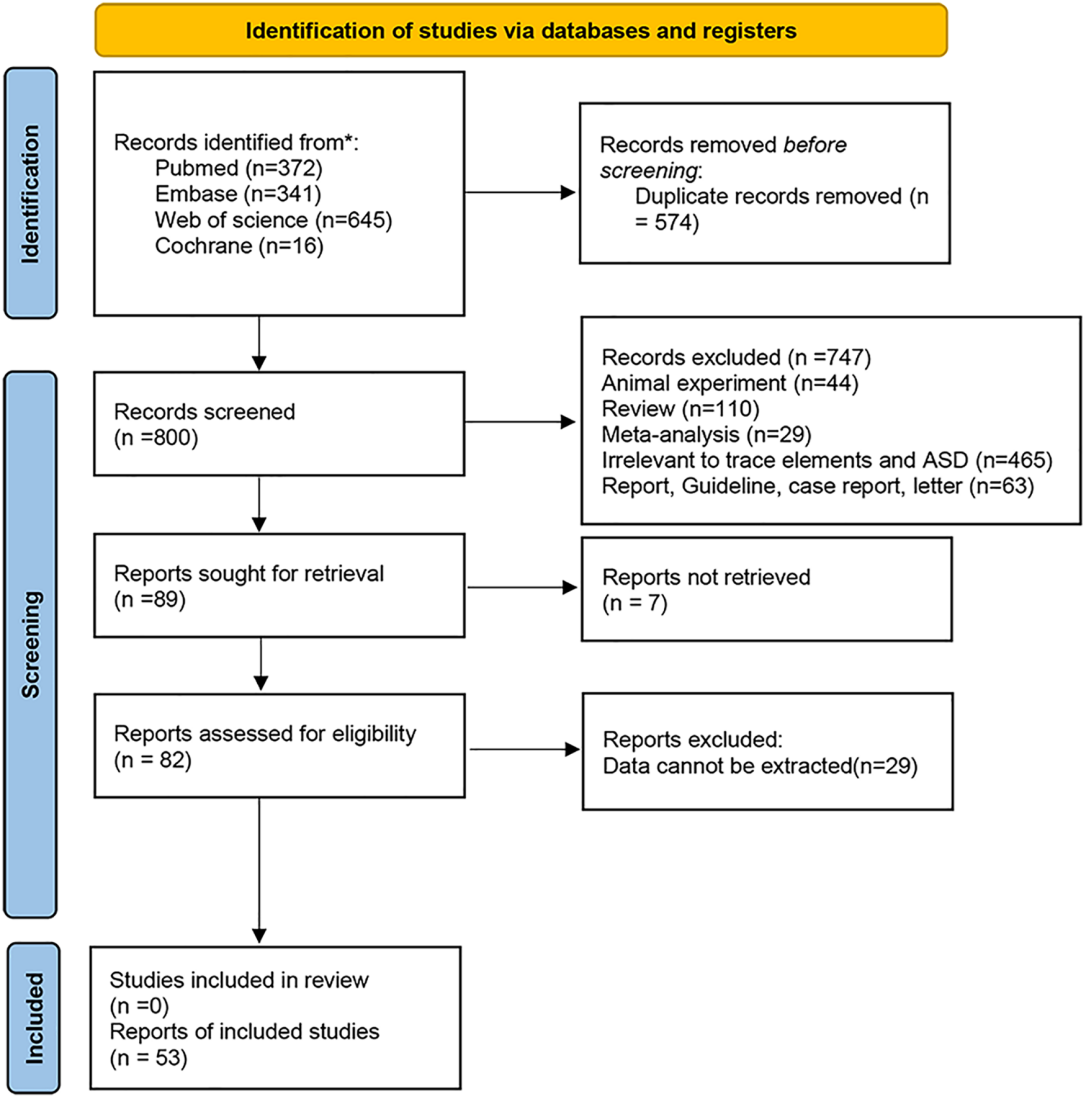
Hg meta-analysis forest plot.

There were 34 studies reporting the difference in As concentration between the ASD group and the healthy control group. A random-effects model was applied for meta-analysis (*I*^2 ^= 94.6%, *P* < 0.001). ASD group had higher Hg concentration compared to the healthy control group [SMD = 0.74, 95%CI (0.44, 1.03), *P* < 0.001] (Figure 6). Subgroup analysis based on geographical regions of the participants and the testing sources showed that compared with the healthy controls, ASD patients had higher Hg concentration in hair [SMD = 0.91, 95%CI (0.35, 1.48), *P* < 0.05], blood [SMD = 0.77, 95%CI (0.35, 1.19), *P* < 0.001], urine [SMD = 0.29, 95%CI (0.11, 0.47), *P* < 0.05], fingernails [SMD = 1.22, 95%CI (0.60, 1.83), *P* < 0.05], and teeth [SMD = 0.40, 95%CI (−0.42, 1.21), *P* > 0.05]. As for geographical regions, ASD patients in Asia [SMD = 1.55, 95%CI (0.82, 2.29), *P* < 0.001], Europe [SMD = 0.55, 95%CI (0.24, 0.86), *P* < 0.001], and Africa [SMD = 0.10, 95%CI (−0.16, 0.36), *P* > 0.05] had higher Hg concentration than the healthy controls in these regions, while patients in North America [SMD = −0.02, 95%CI (−0.62, 0.59), *P* > 0.05] had a lower Hg concentration(Table 2). Sensitivity analysis showed that the removal of the included studies one by one did not reverse the results, suggesting the results of the meta-analysis were robust. Significant publication bias was observed (*P* = 0.002 in Begg's test and *P* = 0.008 in Egger’s test) (Figure 3). Trim-and-Fill method showed no newly-added studies, and no small-sample effect. Publication bias did not affect the results (Supplementary Files S4).

## 4. Discussion

Among the included 53 studies, Atomic Absorption Spectrometry (AAS), Inductively Coupled Plasma Mass-spectrometry (ICPMS), Flame Atomic Absorption Spectrometry (FAAS), Automatic Biochemical Analysis (ABA), and Inductively Coupled Plasma Emission Spectrum Analysis (ICPOES) were applied to detect microelements in the samples. The results showed that the ASD group had significantly higher Cd, Pb, arsenic, and Hg concentrations than the healthy control group. Subgroup analysis of testing methods indicated that concentrations of the 4 heavy metals tested by different methods were all higher in the ASD group, which suggested that testing methods were not the source of heterogeneity. Subgroup analysis of testing sources showed that compared with the heavy controls, ASD patients had higher hair and urine concentrations of the 4 heavy metals but had lower urine concentrations of Pb and arsenic. Based on previous studies, this might be associated with the patients’ reduced ability to excrete heavy metals. Subgroup analysis of geographical regions showed that ASD patients in Asian and European countries had higher concentrations of the 4 heavy metals. However, those in North America (especially the United States) had lower concentrations of Cd, arsenic, and Hg. The specific mechanisms remain unclear, leading to heterogeneity among the studies. In general, the results of different testing sources and geographical regions were controversial, which could be the source of heterogeneity.

The mechanisms of heavy metals exposure causing ASD might be related to the following aspects: Heavy metals are believed to play a role in the pathogenesis of ASD through epigenetic mechanisms. Heavy metals exposure during the growth of children could be of potential epigenetic effects on DNA methylation by mediating the dysregulation of methyltransferase (65, 66). In addition, exposure to air pollutants could cause oxidative stress and inflammatory response because heavy metals disrupt the enzyme function and cell signaling processes, produce ROS, and mediate autoimmune responses. ASD patients often have deficiencies in the defense system against ROS and impaired REDOX homeostasis, which increases susceptibility to oxidative stress, leading to consequences such as heavy metal poisoning. This might be related to altered glutathione (GSH) synthesis and impaired antioxidant defense system (6, 67, 68). Lastly, studies have shown that heavy metals could accumulate within the central neuronal system of ASD patients due to the reduced ability to excrete these substances, leading to neurotoxicity (69, 70).

### 4.1. Cd exposure and ASD

Thea S Skogheim et al, have found that individuals exposed to Cd could increase the risk for ASD in their offspring (71). In this meta-analysis, the ASD group had evidently higher Cd concentration than the healthy control group (*P* > 0.05). Subgroup analysis showed that ASD patients had evidently higher Cd concentrations in their hair and blood compared to the healthy controls (*P* > 0.05), while they had a lower urine concentration (*P* > 0.05), which is consistent with the conclusion that ASD patients have a reduced ability of heavy metals excretion. Asian and European patients had higher Cd concentration (*P* > 0.05), whereas North American patients had an opposite result (*P* < 0.05), indicating geographical regions could cause differences in Cd concentration among ASD patients. The specific reason is still unclear and further research is needed in the future.

Cd is a kind of pollutant introduced into the environment due to the rapid development of industry and modern technology. People are mainly exposed to Cd from water, food, and air pollutants through respiratory absorption, and this substance could accumulate within the body, with a half-life period of 25–30 years (72). Cd is ranked seventh in the Substance Priority List released by the U.S. Agency for Toxic Substances and Disease Registry (ATSDR) (73), indicating its adverse impact on the human body. The kidney and liver would be the biggest victims, and 50% of the absorbed Cd is deposited in these organs. Therefore, they are the most susceptible to the adverse impact of Cd exposure (74). Cd damage to the central nervous system, especially the immature brain, could lead to neurodysplasia, memory deterioration, and hypophrenia (75). 70% of ASD patients have intellectual disability. Its association with early exposure to Cd still needs to be further discussed. The findings of this study support the need to reduce the consumption of Cd, especially in children whose brains are still growing.

### 4.2. Pb exposure and ASD

Pb is a kind of non-ferrous heavy metal element that naturally exists in the earth crust. It is abundant in the environment. Even a very low concentration of Pb could be of irreversible neurotoxicity. Frequent human exposure to Pb has been a severe global environmental health problem (59). In this meta-analysis, the ASD group had a significantly higher Pb concentration than the healthy control group (*P* < 0.001). In the subgroup analysis of geographical regions, the ASD group also had a higher Pb concentration than the healthy control group, in which the differences between Asia and Europe were statistically significant (*P* < 0.001). Subgroup analysis of testing sources found that ASD patients had significantly higher hair and blood Pb concentrations than the healthy controls (*P* < 0.001), while had a lower urine concentration (*P* > 0.05), which is consistent with the study results by Nakhaee S et al. Their findings also support that ASD patients have a declined heavy metals (including Pb) excretion ability (16, 69, 70, 76, 77). The reason for this declined ability might be, according to previous studies, that ASD patients typically have reduced levels of anti-oxidants, and that the excretion of calcium ions (Ca^2+^) could competitively inhibit that Pb excretion, leading to reduced binding of Pb to the anti-oxidants and subsequently a low urine Pb excretion rate (21). Unlike other heavy metals, Pb could directly damage human brain cells, especially the neural plate of fetus. Fetal Pb exposure obscures the cerebral sulci and causes wide neurodysplasia and hypophrenia (78). ASD is a neurodevelopmental disorder, characterized by extensively abnormal neural network connections. The results of this study support that Pb is associated with the occurrence of ASD. Given the neurotoxicity of Pb, our findings would be of directive significance for clinical practice. Daily exposure to Pb should be avoided, especially for pregnant women and children.

### 4.3. Arsenic exposure and ASD

Arsenic is a prevalent toxic substance that widely exists in groundwater all over the world. It is mainly absorbed through the digestive tract and respiratory system. Arsenic compounds are capable of inhibiting the activity of more than 200 enzymes within the human body, causing damage to the structure and function of the central nervous system. Moreover, arsenic could disrupt cellular metabolism (54, 79). Extensive exposure to inorganic arsenic could occur through drinking polluted water, industrial production, consumption of rice-based foods, and cigarettes and tobacco (80, 81). Emerging epidemiological investigations support that arsenic exposure especially during the critical period of neurodevelopment could be an environmental risk factor for ASD occurrence (82, 83).

This meta-analysis found that the ASD group had a higher arsenic concentration than the healthy control group (*P* < 0.001). Subgroup analysis of testing sources showed that compared with the healthy controls, ASD children had higher arsenic concentrations in their hair, urine, and blood (*P* < 0.05), which further validated the hypothesis that arsenic exposure might be associated with the occurrence of ASD. ASD was once considered to be a metabolic disease, characterized by a metabolic disorder of glucose, lipid, and amino acid (84, 85). Arsenic could affect the cellular metabolic processes (86).

The results of this study support the importance of reducing arsenic exposure. It remains to be further explored the specific mechanisms of the arsenic concentration increase in ASD patients and the association between arsenic exposure and the metabolic disturbance of ASD patients.

### 4.4. Hg exposure and ASD

Hg is a toxic heavy metal substance, which is methylated in the air or water, accumulates in animal tissues, and deposits in the human body through the food chain. High levels of Hg exposure could induce irreversible neuronal and renal injury (73). Meta-analysis showed that ASD children had a significantly higher Hg concentration than the healthy controls (*P* < 0.001). Subgroup analysis indicated that ASD children had significantly higher Hg concentrations in their hair, blood, and urine, compared with the healthy control group (*P* < 0.05). The patients in Asia and Europe had a higher Hg concentration than the healthy controls (*P* < 0.001), while those in North America had a lower concentration (*P* > 0.05). A possible reason for the higher Hg concentration in ASD patients is that they take antibiotics more frequently than normal people. An animal experiment has shown that antibiotics can block the excretion of Hg, reduce the number of intestinal flora by demethylating methylmercury, and possibly increase the number of yeasts and escherichia coli by methylating inorganic mercury, thereby promoting mercury uptake and lowering mercury excretion (87). On the other hand, a lower level of glutathione and higher level of oxidative stress in ASD patients can also compromise mercury excretion, resulting in a heavier burden on the body (20). The present study demonstrates that it is necessary to reduce exposure to Hg, especially for pregnant women and children in brain-developing.

### 4.5. Strengths and limitations

The strength of this study lies in that: Firstly, we selected 4 heavy metals that are the most controversial and the most studied, and performed a comprehensive search for the published studies. The results of this study are reported in accordance with the PRISMA guideline, and most of the included studies are the latest published, with high quality. Secondly, our statistical analysis strategy is relatively conservative, including sensitivity analysis, subgroup analysis, and publication bias assessment, to assess the potential effects of different geographical regions and different testing sources on the results. We have observed that due to the declined heavy metal excretion ability in ASD patients, the results are different in their urine specimens compared to their hair and blood samples. In addition, the patients in North America have different results from those in Asia and Europe, leading to the heterogeneity of the results.

This study has several limitations. Firstly, the included studies are case-control design so that a specific causality could not be determined between heavy metals exposure and ASD occurrence. Secondly, all the study results are directly from the level of case group and control group, so the effects of confounding factors (age, gender, BMI, etc.) on the results could not be assessed.

## 5. Conclusion

Compared with the healthy control group, the ASD group had higher concentrations of Cd, Pb, arsenic, and Hg, and the differences in Pb, arsenic, and Hg were statistically significant. Subgroup analysis indicated the results could be of regional difference. Studies in Asia and Europe showed that ASD children had higher concentrations of Cd, Pb, arsenic, and Hg than the healthy controls, while studies in North America yielded the opposite results regarding Cd, arsenic, and Hg. The reason remains unclear. In addition, ASD children had higher concentrations of the 4 heavy metals in their hair and blood, while had lower concentrations of Cd and Pb in urine, which could be attributed to their declined heavy metal excretion ability. Future studies need to focus on: (1) Biological mechanisms of heavy metals exposure inducing ASD. (2) The regional difference of this association. In general, reducing heavy metals exposure and keeping a good diet would be beneficial to ASD prevention.

## Data Availability

The original contributions presented in the study are included in the article/**Supplementary Material**, further inquiries can be directed to the corresponding authors.

## Supplementary material

SUPPLEMENTARY FILES 1

Search strategy.

SUPPLEMENTARY FILES 2

Pb published offset analysis.

SUPPLEMENTARY FILES 3

Pb published offset analysis.

SUPPLEMENTARY FILES 4

Hg published offset analysis.
