# Supplementary material for: Association between heavy metals exposure (cadmium, lead, arsenic, mercury) and child autistic disorder: a systematic review and meta-analysis
Source: Front Pediatr. 2023 Jul 4;11:1169733. doi: 10.3389/fped.2023.1169733 (PMC10353844; doi:10.3389/fped.2023.1169733)
Supplement: Supplementary file 1 [file Datasheet1.pdf]

Supplementary Files 1 Search strategy

1 Pubmed

Search number	Query	Sort By	Filters	Search Details	Results
16	((((("Autistic Disorder"[Mesh]) OR (Autism Spectrum Disorder[MeSH])) OR ((((((((((((((((Autism Spectrum Disorder[Title/Abstract]) OR (Autistic Disorder[Title/Abstract])) OR (Autism Spectrum Disorders[Title/Abstract])) OR (Autistic Spectrum Disorder[Title/Abstract])) OR (Autistic Spectrum Disorders[Title/Abstract])) OR (Disorder, Autistic Spectrum[Title/Abstract])) OR (Disorder, Autistic[Title/Abstract])) OR (Disorders, Autistic[Title/Abstract])) OR (Kanner's Syndrome[Title/Abstract])) OR (Kanner Syndrome[Title/Abstract])) OR (Kanners Syndrome[Title/Abstract])) OR (Autism, Infantile[Title/Abstract])) OR (Infantile Autism[Title/Abstract])) OR (Autism[Title/Abstract])) OR (Autism, Early Infantile[Title/Abstract])) OR (Early Infantile Autism[Title/Abstract])) OR (Infantile Autism, Early[Title/Abstract]))) AND ((child) OR (Children)))) AND (((("Metals, Heavy"[Mesh]) OR ("Trace Elements"[Mesh])) OR ((((((((((trace elements[Title/Abstract]) OR (Trace Element[Title/Abstract])) OR (Element, Trace[Title/Abstract])) OR (Elements, Trace[Title/Abstract])) OR (Biometals[Title/Abstract])) OR (Biometal[Title/Abstract])) OR (Trace Minerals[Title/Abstract])) OR (Mineral, Trace[Title/Abstract])) OR (Minerals, Trace[Title/Abstract])) OR (Trace Mineral[Title/Abstract]))) OR ((((Cadmium[Title/Abstract]) OR (plumbum[Title/Abstract])) OR (arsenic[Title/Abstract])) OR (Mercury[Title/Abstract])))			("Autistic Disorder"[MeSH Terms] OR "autism spectrum disorder"[MeSH Terms] OR ("autism spectrum disorder"[Title/Abstract] OR "Autistic Disorder"[Title/Abstract] OR "autism spectrum disorders"[Title/Abstract] OR "autistic spectrum disorder"[Title/Abstract] OR "autistic spectrum disorders"[Title/Abstract] OR "disorder autistic spectrum"[Title/Abstract] OR "disorder autistic"[Title/Abstract] OR "disorders autistic"[Title/Abstract] OR "kanner s syndrome"[Title/Abstract] OR "kanner syndrome"[Title/Abstract] OR ("Kanners"[All Fields] AND "Syndrome"[Title/Abstract]) OR "autism infantile"[Title/Abstract] OR "infantile autism"[Title/Abstract] OR "Autism"[Title/Abstract] OR "autism early infantile"[Title/Abstract] OR "early infantile autism"[Title/Abstract] OR "infantile autism early"[Title/Abstract])) AND ("child"[MeSH Terms] OR "child"[All Fields] OR "children"[All Fields] OR "child s"[All Fields] OR "children s"[All Fields] OR "childrens"[All Fields] OR "childs"[All Fields] OR ("child"[MeSH Terms] OR "child"[All Fields] OR "children"[All Fields] OR "child s"[All Fields] OR "children s"[All Fields] OR "childrens"[All Fields] OR "childs"[All Fields])) AND ("metals, heavy"[MeSH Terms] OR "Trace Elements"[MeSH Terms] OR ("Trace Elements"[Title/Abstract] OR "trace element"[Title/Abstract] OR "element trace"[Title/Abstract] OR "elements trace"[Title/Abstract] OR "Biometals"[Title/Abstract] OR "Biometal"[Title/Abstract] OR "trace minerals"[Title/Abstract] OR "mineral trace"[Title/Abstract] OR "minerals trace"[Title/Abstract] OR "trace	372

		mineral"[Title/Abstract]) OR ("Cadmium"[Title/Abstract] OR "plumbum"[Title/Abstract] OR "arsenic"[Title/Abstract] OR "Mercury"[Title/Abstract]))	
15	(((((("Autistic Disorder"[Mesh]) OR (Autism Spectrum Disorder[MeSH])) OR (((((((((((((((Autism Spectrum Disorder[Title/Abstract]) OR (Autistic Disorder[Title/Abstract])) OR (Autism Spectrum Disorders[Title/Abstract])) OR (Autistic Spectrum Disorder[Title/Abstract])) OR (Autistic Spectrum Disorders[Title/Abstract])) OR (Disorder, Autistic Spectrum[Title/Abstract])) OR (Disorder, Autistic[Title/Abstract])) OR (Disorders, Autistic[Title/Abstract])) OR (Kanner's Syndrome[Title/Abstract])) OR (Kanner Syndrome[Title/Abstract])) OR (Kanners Syndrome[Title/Abstract])) OR (Autism, Infantile[Title/Abstract])) OR (Infantile Autism[Title/Abstract])) OR (Autism[Title/Abstract])) OR (Autism, Early Infantile[Title/Abstract])) OR (Early Infantile Autism[Title/Abstract])) OR (Infantile Autism, Early[Title/Abstract]))) AND ((child) OR (Children))) AND ((("Metals, Heavy"[Mesh]) OR ("Trace Elements"[Mesh])) OR ((((((((((trace elements[Title/Abstract]) OR (Trace Element[Title/Abstract])) OR (Element, Trace[Title/Abstract])) OR (Elements, Trace[Title/Abstract])) OR (Biometals[Title/Abstract])) OR (Biometal[Title/Abstract])) OR (Trace Minerals[Title/Abstract])) OR (Mineral, Trace[Title/Abstract])) OR (Minerals, Trace[Title/Abstract])) OR (Trace Mineral[Title/Abstract])))) AND ((((Cadmium[Title/Abstract]) OR (plumbum[Title/Abstract])) OR (arsenic[Title/Abstract])) OR (Mercury[Title/Abstract]))) AND (((case-control study[Title/Abstract]) OR (Randomized Controlled Trials as Topic[Title/Abstract])) OR (Cohort Studies[Title/Abstract]))	("Autistic Disorder"[MeSH Terms] OR "autism spectrum disorder"[MeSH Terms] OR ("autism spectrum disorder"[Title/Abstract] OR "Autistic Disorder"[Title/Abstract] OR "autism spectrum disorders"[Title/Abstract] OR "autistic spectrum disorder"[Title/Abstract] OR "autistic spectrum disorders"[Title/Abstract] OR "disorder autistic spectrum"[Title/Abstract] OR "disorder autistic"[Title/Abstract] OR "disorders autistic"[Title/Abstract] OR "kanner s syndrome"[Title/Abstract] OR "kanner syndrome"[Title/Abstract] OR ("Kanners"[All Fields] AND "Syndrome"[Title/Abstract]) OR "autism infantile"[Title/Abstract] OR "infantile autism"[Title/Abstract] OR "Autism"[Title/Abstract] OR "autism early infantile"[Title/Abstract] OR "early infantile autism"[Title/Abstract] OR "infantile autism early"[Title/Abstract])) AND ("child"[MeSH Terms] OR "child"[All Fields] OR "children"[All Fields] OR "child s"[All Fields] OR "children s"[All Fields] OR "childrens"[All Fields] OR "childs"[All Fields] OR ("child"[MeSH Terms] OR "child"[All Fields] OR "children"[All Fields] OR "child s"[All Fields] OR "children s"[All Fields] OR "childrens"[All Fields] OR "childs"[All Fields])) AND ("metals, heavy"[MeSH Terms] OR "Trace Elements"[MeSH Terms] OR ("Trace Elements"[Title/Abstract] OR "trace element"[Title/Abstract] OR "element trace"[Title/Abstract] OR "elements trace"[Title/Abstract] OR "Biometals"[Title/Abstract] OR "Biometal"[Title/Abstract] OR "trace minerals"[Title/Abstract] OR "mineral trace"[Title/Abstract] OR "minerals	12

			trace"[Title/Abstract] OR "trace mineral"[Title/Abstract])) AND ("Cadmium"[Title/Abstract] OR "plumbum"[Title/Abstract] OR "arsenic"[Title/Abstract] OR "Mercury"[Title/Abstract]) AND ("case control study"[Title/Abstract] OR "randomized controlled trials as topic"[Title/Abstract] OR "cohort studies"[Title/Abstract])	
14	((case-control study[Title/Abstract]) OR (Randomized Controlled Trials as Topic[Title/Abstract])) OR (Cohort Studies[Title/Abstract])		"case control study"[Title/Abstract] OR "randomized controlled trials as topic"[Title/Abstract] OR "cohort studies"[Title/Abstract]	139,550
13	"Cohort Studies"[Mesh]	Most Recent	"Cohort Studies"[MeSH Terms]	2,397,257
12	((("Metals, Heavy"[Mesh]) OR ("Trace Elements"[Mesh])) OR (((((((((trace elements[Title/Abstract]) OR (Trace Element[Title/Abstract])) OR (Element, Trace[Title/Abstract])) OR (Elements, Trace[Title/Abstract])) OR (Biometals[Title/Abstract])) OR (Biometal[Title/Abstract])) OR (Trace Minerals[Title/Abstract])) OR (Mineral, Trace[Title/Abstract])) OR (Minerals, Trace[Title/Abstract])) OR (Trace Mineral[Title/Abstract]))) OR ((((Cadmium[Title/Abstract]) OR (plumbum[Title/Abstract])) OR (arsenic[Title/Abstract])) OR (Mercury[Title/Abstract]))		"metals, heavy"[MeSH Terms] OR "Trace Elements"[MeSH Terms] OR "Trace Elements"[Title/Abstract] OR "trace element"[Title/Abstract] OR "element trace"[Title/Abstract] OR "elements trace"[Title/Abstract] OR "Biometals"[Title/Abstract] OR "Biometal"[Title/Abstract] OR "trace minerals"[Title/Abstract] OR "mineral trace"[Title/Abstract] OR "minerals trace"[Title/Abstract] OR "trace mineral"[Title/Abstract] OR "Cadmium"[Title/Abstract] OR "plumbum"[Title/Abstract] OR "arsenic"[Title/Abstract] OR "Mercury"[Title/Abstract]	694,044
11	(((Cadmium[Title/Abstract]) OR (plumbum[Title/Abstract])) OR (arsenic[Title/Abstract])) OR (Mercury[Title/Abstract])		"Cadmium"[Title/Abstract] OR "plumbum"[Title/Abstract] OR "arsenic"[Title/Abstract] OR "Mercury"[Title/Abstract]	114,021
10	(("Metals, Heavy"[Mesh]) OR ("Trace Elements"[Mesh])) OR (((((((((trace elements[Title/Abstract]) OR (Trace Element[Title/Abstract])) OR (Element, Trace[Title/Abstract])) OR (Elements, Trace[Title/Abstract])) OR (Biometals[Title/Abstract])) OR (Biometal[Title/Abstract])) OR (Trace Minerals[Title/Abstract])) OR (Mineral, Trace[Title/Abstract])) OR (Minerals, Trace[Title/Abstract])) OR (Trace Mineral[Title/Abstract]))		"metals, heavy"[MeSH Terms] OR "Trace Elements"[MeSH Terms] OR "Trace Elements"[Title/Abstract] OR "trace element"[Title/Abstract] OR "element trace"[Title/Abstract] OR "elements trace"[Title/Abstract] OR "Biometals"[Title/Abstract] OR "Biometal"[Title/Abstract] OR "trace minerals"[Title/Abstract] OR "mineral trace"[Title/Abstract] OR "minerals trace"[Title/Abstract] OR "trace mineral"[Title/Abstract]	638,376

9	((((((((trace elements[Title/Abstract]) OR (Trace Element[Title/Abstract])) OR (Element, Trace[Title/Abstract])) OR (Elements, Trace[Title/Abstract])) OR (Biometals[Title/Abstract])) OR (Biometal[Title/Abstract])) OR (Trace Minerals[Title/Abstract])) OR (Mineral, Trace[Title/Abstract])) OR (Minerals, Trace[Title/Abstract])) OR (Trace Mineral[Title/Abstract])		"trace elements"[Title/Abstract] OR "trace element"[Title/Abstract] OR "element trace"[Title/Abstract] OR "elements trace"[Title/Abstract] OR "Biometals"[Title/Abstract] OR "Biometal"[Title/Abstract] OR "trace minerals"[Title/Abstract] OR "mineral trace"[Title/Abstract] OR "minerals trace"[Title/Abstract] OR "trace mineral"[Title/Abstract]	24,543
8	"Trace Elements"[Mesh]	Most Recent	"Trace Elements"[MeSH Terms]	19,108
7	"Metals, Heavy"[Mesh]	Most Recent	"metals, heavy"[MeSH Terms]	618,182
6	(child) OR (Children)		"child"[MeSH Terms] OR "child"[All Fields] OR "children"[All Fields] OR "child s"[All Fields] OR "children s"[All Fields] OR "childrens"[All Fields] OR "childs"[All Fields] OR "child"[MeSH Terms] OR "child"[All Fields] OR "children"[All Fields] OR "child s"[All Fields] OR "children s"[All Fields] OR "childrens"[All Fields] OR "childs"[All Fields]	3,018,196
5	"Child"[Mesh]	Most Recent	"Child"[MeSH Terms]	2,100,547
4	(("Autistic Disorder"[Mesh]) OR (Autism Spectrum Disorder[MeSH])) OR ((((((((((((((((Autism Spectrum Disorder[Title/Abstract]) OR (Autistic Disorder[Title/Abstract])) OR (Autism Spectrum Disorders[Title/Abstract])) OR (Autistic Spectrum Disorder[Title/Abstract])) OR (Autistic Spectrum Disorders[Title/Abstract])) OR (Disorder, Autistic Spectrum[Title/Abstract])) OR (Disorder, Autistic[Title/Abstract])) OR (Disorders, Autistic[Title/Abstract])) OR (Kanner's Syndrome[Title/Abstract])) OR (Kanner Syndrome[Title/Abstract])) OR (Kanners Syndrome[Title/Abstract])) OR (Autism, Infantile[Title/Abstract])) OR (Infantile Autism[Title/Abstract])) OR (Autism[Title/Abstract])) OR (Autism, Early Infantile[Title/Abstract])) OR (Early Infantile Autism[Title/Abstract])) OR (Infantile Autism, Early[Title/Abstract]))		"Autistic Disorder"[MeSH Terms] OR "autism spectrum disorder"[MeSH Terms] OR ("autism spectrum disorder"[Title/Abstract] OR "Autistic Disorder"[Title/Abstract] OR "autism spectrum disorders"[Title/Abstract] OR "autistic spectrum disorder"[Title/Abstract] OR "autistic spectrum disorders"[Title/Abstract] OR "disorder autistic spectrum"[Title/Abstract] OR "disorder autistic"[Title/Abstract] OR "disorders autistic"[Title/Abstract] OR "kanner s syndrome"[Title/Abstract] OR "kanner syndrome"[Title/Abstract] OR ("Kanners"[All Fields] AND "Syndrome"[Title/Abstract]) OR "autism infantile"[Title/Abstract] OR "infantile autism"[Title/Abstract] OR "Autism"[Title/Abstract] OR "autism early infantile"[Title/Abstract] OR "early infantile autism"[Title/Abstract]	63,934

			OR "infantile autism early"[Title/Abstract])	
3	(((((((((((((((Autism Spectrum Disorder[Title/Abstract]) OR (Autistic Disorder[Title/Abstract])) OR (Autism Spectrum Disorders[Title/Abstract])) OR (Autistic Spectrum Disorder[Title/Abstract])) OR (Autistic Spectrum Disorders[Title/Abstract])) OR (Disorder, Autistic Spectrum[Title/Abstract])) OR (Disorder, Autistic[Title/Abstract])) OR (Disorders, Autistic[Title/Abstract])) OR (Kanner's Syndrome[Title/Abstract])) OR (Kanner Syndrome[Title/Abstract])) OR (Kanners Syndrome[Title/Abstract])) OR (Autism, Infantile[Title/Abstract])) OR (Infantile Autism[Title/Abstract])) OR (Autism[Title/Abstract])) OR (Autism, Early Infantile[Title/Abstract])) OR (Early Infantile Autism[Title/Abstract])) OR (Infantile Autism, Early[Title/Abstract])		"autism spectrum disorder"[Title/Abstract] OR "autistic disorder"[Title/Abstract] OR "autism spectrum disorders"[Title/Abstract] OR "autistic spectrum disorder"[Title/Abstract] OR "autistic spectrum disorders"[Title/Abstract] OR "disorder autistic spectrum"[Title/Abstract] OR "disorder autistic"[Title/Abstract] OR "disorders autistic"[Title/Abstract] OR "kanner s syndrome"[Title/Abstract] OR "kanner syndrome"[Title/Abstract] OR ("Kanners"[All Fields] AND "Syndrome"[Title/Abstract]) OR "autism infantile"[Title/Abstract] OR "infantile autism"[Title/Abstract] OR "Autism"[Title/Abstract] OR "autism early infantile"[Title/Abstract] OR "early infantile autism"[Title/Abstract] OR "infantile autism early"[Title/Abstract]	58,251
2	Autism Spectrum Disorder[MeSH]		"autism spectrum disorder"[MeSH Terms]	39,607
1	"Autistic Disorder"[Mesh]	Most Recent	"Autistic Disorder"[MeSH Terms]	23,945

2. Embase

No.	Query	Results
#13	#3 AND #6 AND #11	341
#11	#9 OR #10	179946
#10	'trace elements':ab,ti OR 'heavy metals':ab,ti OR cadmium:ab,ti OR plumbum:ab,ti OR arsenic:ab,ti OR mercury:ab,ti	179941
#9	#7 OR #8	63799
#8	'trace elements':ab,ti OR 'heavy metals':ab,ti	63792
#7	'heavy metals'/exp	12
#6	#4 OR #5	3673401
#5	child:ab,ti OR children:ab,ti	1859069
#4	'child'/exp	3233031
#3	#1 OR #2	98044
#2	autism:ab,ti OR 'autism spectrum disorder':ab,ti OR 'kanner syndrome':ab,ti OR 'autistic disorder':ab,ti	71624
#1	'autism'/exp	90264

3. WOS

Search number	Query	Sort By	Filters	Search Details	Results
1	**autism** (Topic)				111640
2	**autism (Topic) or Autistic Disorder (Topic) or Autism Spectrum Disorder (Topic) or Kanner Syndrome (Topic) or Kanner's Syndrome (Topic)**				119048
3	**child** (Topic) or **children** (Topic)				4041620
4	**heavy metals** (Topic) or **trace elements** (Topic) or **Trace Minerals** (Topic) or **Cadmium** (Topic) or **plumbum** (Topic) or **arsenic** (Topic) or **Mercury** (Topic)				1155354
5	**#13 AND #15 AND #16**				645

4. Cochrane

Search number	Query	Sort By	Filters	Search Details	Results
1	MeSH descriptor: [Autistic Disorder] explode all trees				1195
2	(Kanners Syndrome):ti,ab,kw OR (autism spectrum disorder):ti,ab,kw OR (Autism):ti,ab,kw OR (autistic disorder):ti,ab,kw OR (Kanner's Syndrome):ti,ab,kw				4530
3	#1 or #2				4530
4	MeSH descriptor: [Child] explode all trees				61990
5	(Child):ti,ab,kw OR (Children):ti,ab,kw				166622
6	#4 or #5				166622
7	MeSH descriptor: [Metals, Heavy] explode all trees				7859
8	(Metals, Heavy):ti,ab,kw OR (Trace Elements):ti,ab,kw				963
9	#7 or #8				8556
10	(Cadmium):ti,ab,kw OR (plumbum):ti,ab,kw OR (arsenic):ti,ab,kw OR (Mercury):ti,ab,kw				1830
11	#9 or #10				10211
12	#3 and #6 and #11				16

Supplementary Files 2 Pb published offset analysis

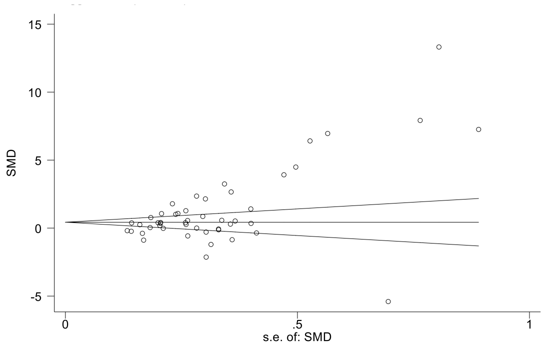

Pb Begg's Test（p= 0.002 < 0.05） showed publication bias

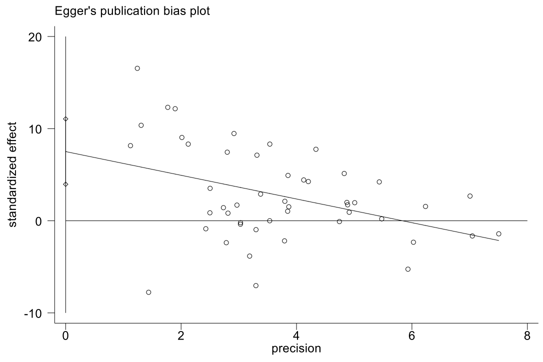

Pb Egger's Test（p= 0.000 < 0.05） showed publication bias

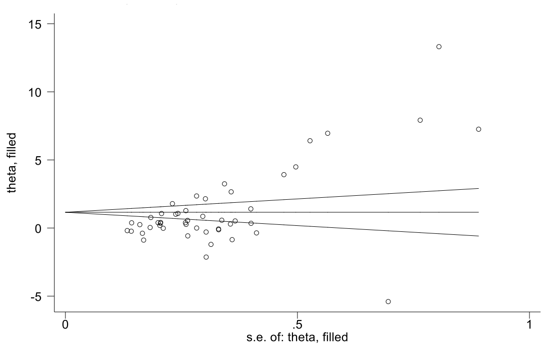

The subtractive complementary method does not add new literature and there are no small sample studies, indicating that publication bias does not affect the final results

Supplementary Files 3 As published offset analysis

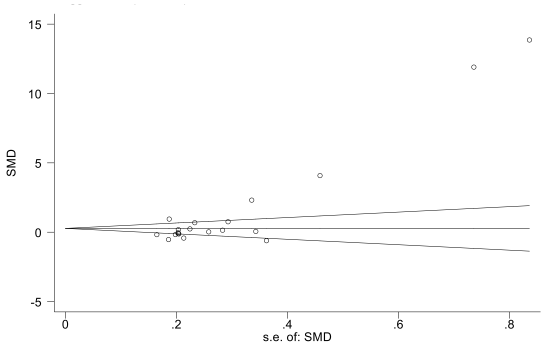

AS Begg's Test（p= 0.003 < 0.05）showed publication bias

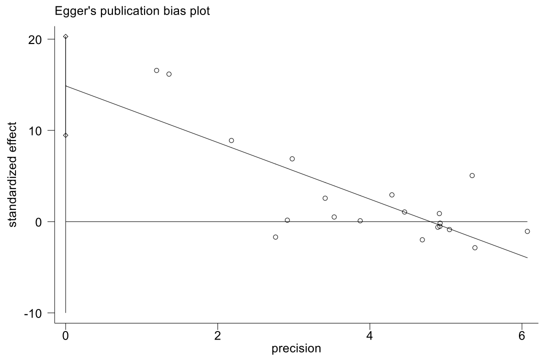

AS Egger's Test（p= 0.000 < 0.05）showed publication bias

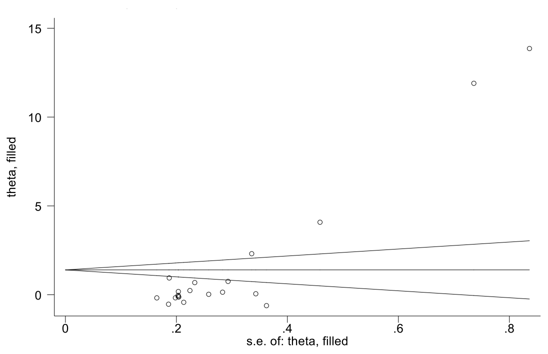

The subtractive complementary method does not add new literature and there are no small sample studies, indicating that publication bias does not affect the final results

Supplementary Files 4 Hg published offset analysis

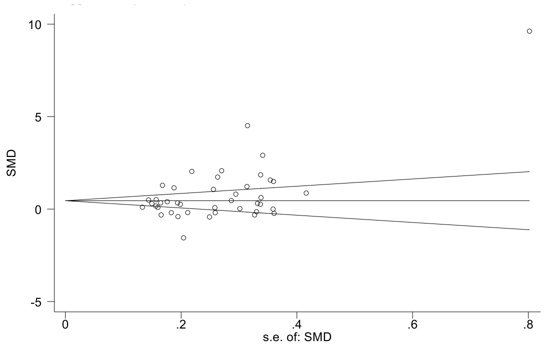

Hg Begg's Test（p= 0.0024 < 0.05） showed publication bias

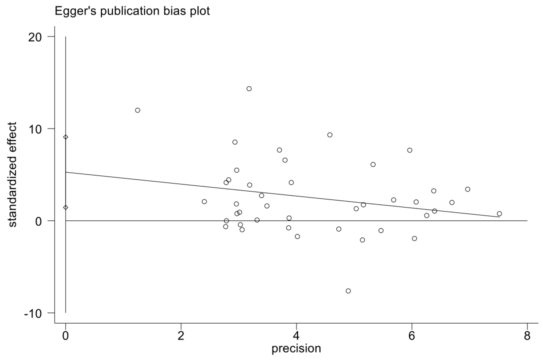

Hg egger's Test（p= 0.008 < 0.05） showed publication bias

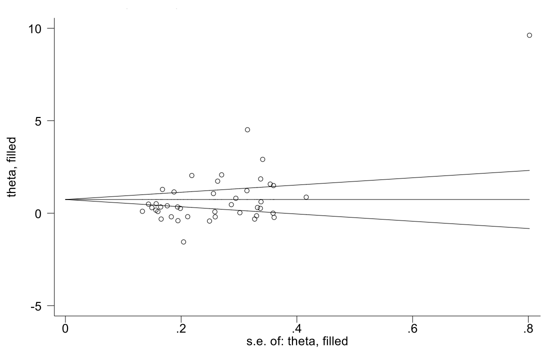

The subtractive complementary method does not add new literature and there are no small sample studies, indicating that publication bias does not affect the final results
